# Multisession CyberKnife Radiosurgery for Advanced Follicular Thyroid Cancer

**DOI:** 10.7759/cureus.6159

**Published:** 2019-11-14

**Authors:** Yuko Harada, Shinichiro Miyazaki

**Affiliations:** 1 Internal Medicine, Harada Naika Clinic, Kawasaki, JPN; 2 Radiation Oncology, Shin-Yurigaoka General Hospital, Kawasaki, JPN

**Keywords:** cyberknife, sbrt, follicular thyroid cancer

## Abstract

Follicular thyroid cancer is a slow-progressing cancer with better prognosis than other types of thyroid cancer. However, treatment for metastatic cancer is very difficult. We treated 11 patients of advanced follicular thyroid cancer with multiple metastases with stereotactic body radiotherapy using the CyberKnife. Of the total 83 lesions that we treated, 53 had complete remission (64%), 28 had partial remission (34%), and two had progressive disease (2%). The complications were minimal. For those patients not indicated for chemotherapy, multisession CyberKnife radiosurgery is a viable option for the treatment of post-surgery recurrent or metastatic follicular thyroid cancer.

## Introduction

Follicular thyroid cancer is classified as differentiated thyroid cancer, which progresses relatively slowly with better prognosis compared to undifferentiated thyroid carcinoma. According to the American Cancer Society, overall five-year survival rates for papillary thyroid carcinoma, follicular thyroid carcinoma, medullary thyroid cancer, and anaplastic thyroid cancer are near 100%, 97%, 90%, and 7%, respectively [1]. Distant metastases occur in patients with differentiated thyroid carcinoma with a prevalence of up to 10% [2]. The aim of treatment is not only to eradicate cancer but also to minimize treatment-related morbidity.

The first choice of treatment is surgery which is difficult for metastatic lesions. Tyrosine kinase inhibitors and chemotherapy are used, but the results are not satisfactory due to harmful side effects [3]. Multisession CyberKnife radiosurgery is less invasive compared to standard surgery, and thereby is more suitable for the elderly patients. Recurrence and metastatic lesions can be expected to be controlled by CyberKnife with minimal complications.

## Materials and methods

From 2013 to 2018, 11 patients (five males, six females) with advanced follicular thyroid cancer with multiple metastasis or recurrence were involved in this study. Multisession stereotactic radiosurgery was performed using the CyberKnife G4 system with the skull-tracking or X-sight spine tracking method. Thin-slice CT and positron emission tomography/CT were performed for CyberKnife treatment planning and follow-up studies. All patients had a follow-up CT scan every three months after the treatment.

The patients’ ages ranged from 43 to 93 years (median 66). The treatments were performed on an outpatient basis. Follow-up periods ranged from 6 to 60 months.

## Results

Most of the recurrent or metastatic lesion regions were spine, lumbar, ribs, scapula, and pelvis. The other lesions were skull, lungs, pleura, lymph nodes, sternum, pituitary, and clavicle. Size of lesions, radiation fractions, and radiation doses are shown in Table 1. Sizes of tumor ranged from 0.48 cc to 500.7 cc. Fractions ranged from 2 to 10. Radiation dose ranged from 1,800 cGy to 5,000 cGy.

**Table 1:** A total of 83 lesions treated by CyberKnife. All the recurrent or metastatic lesions are shown according to the region and size. The fractions and radiation doses for each lesion are shown.

	Spine			Lumbar			Rib(s)			Scapula			Pelvis			Others		
	Size(cc)	Fr	Dose(cGy)	Size(cc)	Fr	Dose(cGy)	Size(cc)	Fr	Dose(cGy)	Size(cc)	Fr	Dose(cGy)	Size(cc)	Fr	Dose(cGy)	Size(cc)	Fr	Dose(cGy)
	0.48	2	3000	0.8	3	2700	1.4	3	2600	0.8	2	2200	0.6	1	1800	1	4	3000
	0.8	2	2200	1.1	3	3000	1.6	2	2800	3.2	3	2500	1	3	2700	4	3	3000
	0.9	3	2700	3	4	3200	3	3	2700	3.4	3	2500	1.1	2	2600	6.3	5	3500
	1.1	3	3000	3.8	3	2700	4	2	2500	5.9	3	3000	2.4	3	3200	7.1	5	4000
	2.4	5	2500	4.4	3	2700	4.7	5	3000	25.8	4	3200	2.6	3	2800	9.3	10	5200
	3.6	3	2700	5.2	3	2700	5.3	5	4000	28.9	3	2700	3.1	3	3000	11.8	5	3500
	4.1	3	3000	5.5	3	2700	7.3	3	2700				3.5	3	2700	16.3	3	2400
	4.3	5	2500	9.5	5	3300	7.4	3	2700				5.2	3	3000	17.1	5	4000
	6.2	5	2500	15.9	5	3300	10.4	3	2700				7.3	3	2700	19.4	8	4000
	6.4	4	2400	20.3	5	2500	10.6	3	2700				9.2	5	3300	19.6	3	2700
	7.4	5	2700	23.8	3	2100	13.7	5	3500				12.3	3	2550	22.4	10	4800
	14.9	3	2100	29.5	5	2600	24.6	3	2700				12.7	3	2700	36.1	10	4000
	16.1	5	2400	57.4	5	2500	27	5	3500				19.7	3	2700	42	5	5000
	20.1	5	3000	86	5	3000	33.9	5	3100				42.6	3	2700	71.5	7	4200
							34.7	5	3200				53.3	5	3500			
							38.4	3	3000				77.5	5	2600			
							38.4	3	2700				500.7	10	3600			
							51.6	5	3500									
Median	4.3	4	2700	9.5	4	2700	10.6	3	2800	5.9	3	2700	7.3	3	2700	17.1	5	4000
Average	6.3		2621	19		2786	17.7		2978	11.3		2683	44.4		2832	20.3		3807

**Figure 1:**
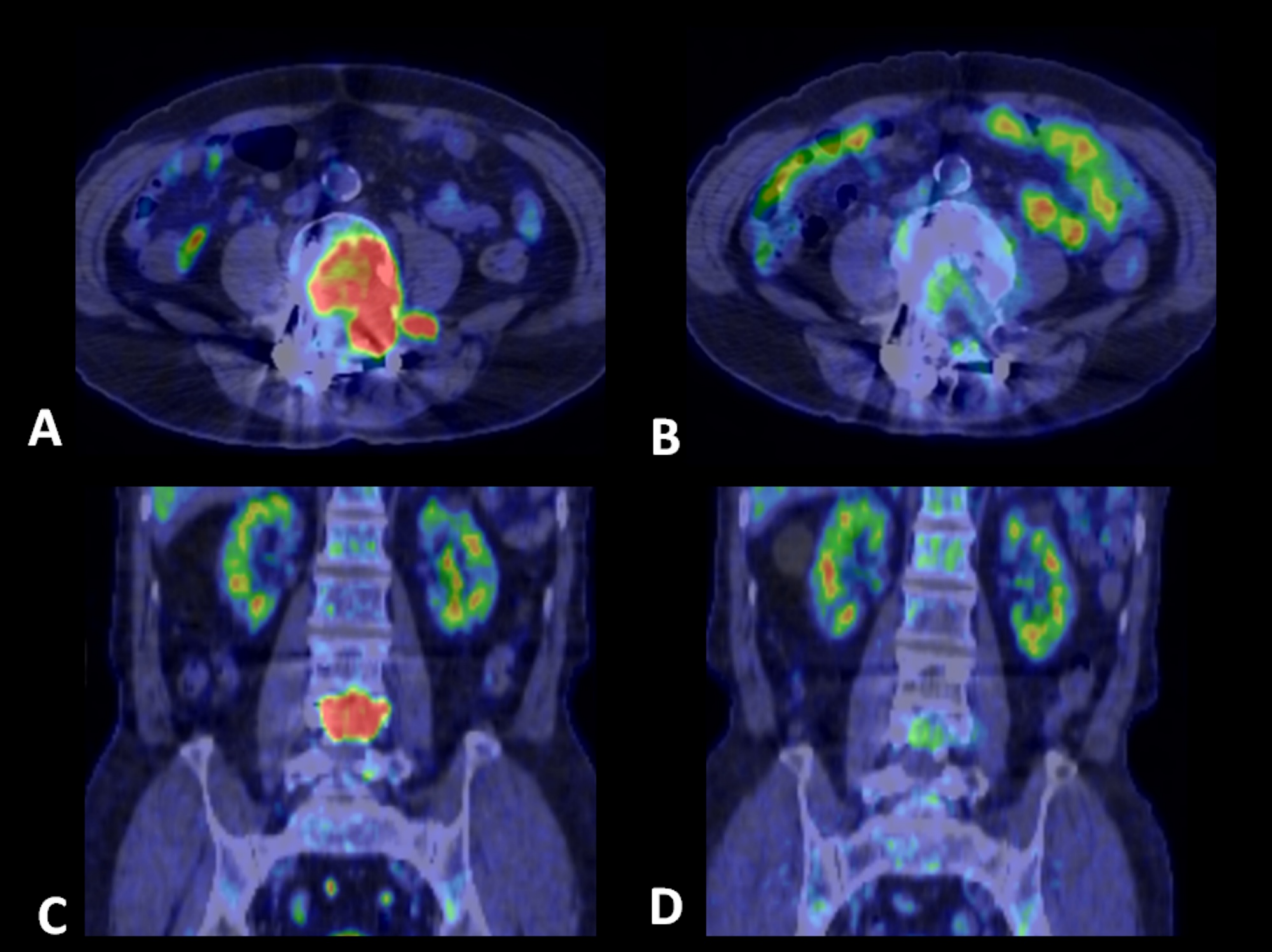
Positron emission tomography/CT scan of lumbar metastasis in a 80-year-old male. A,C: before treatment. B,D: six months after treatment. Fluorodeoxyglucose uptake was remarkably decreased. Tumor targeting volume was 15.9 cc, fraction was 5, and radiation dose was 3,300 cGy.

Typical treatment case is shown in Figure 1. Fluorodeoxyglucose uptake was remarkably decreased six months after the treatment.

Of the total 83 lesions, 53 had complete remission (64%), 28 had partial remission (34%), and 2 (2%) had progressive disease. Complications were minimal, and no toxicity over grade 3 was observed. One patient died of GI bleeding due to thrombocytopenia after chemotherapy with lenvatinib which she received after CyberKnife, but she had no complication of stereotactic body radiotherapy (SBRT) for six months before the chemotherapy. All the other patients are alive and enjoying their normal lives.

## Discussion

Treatment strategy for advanced follicular thyroid cancer is difficult. Radioiodine therapy with I-131 (RIT) can be used for the treatment of radioiodine-positive tumor residues, lymph node, and distant metastases with curative or palliative intention [2]. RIT is generally well tolerated, but causes some side effects such as thyroiditis, gastritis, hypospermia, and bone marrow depression [2,4]. For cancer with low radioiodine uptake, RIT is not a therapeutic option.

Chemotherapy has shown poor outcome for advanced or metastatic thyroid cancer [5]. Thyrosine kinase inhibitors are a new approach to systemic therapy. A recent review on this therapy showed progression-free survival of 10 to 18 months, but it may have severe side effects such as hypertension, vasoconstriction, diarrhea, nausea, and hepatotoxicity [6].

The CyberKnife system is a non-invasive treatment for tumors where radiation therapy is indicated. It can be an alternative to standard surgery for the patients who are contraindicated to surgery. It is a robotic radiation delivery system that delivers a maximum dose of radiation precisely to the tumor from many different angles.

There are only two studies describing SBRT with CyberKnife for thyroid cancer. Kawabe et al. reported a case of recurrent lymph node metastasis of papillary thyroid carcinoma successfully treated with CyberKnife [7]. Ishigaki et al. reported successful SBRT by CyberKnife for 52 locoregional recurrent lesions in 31 patients with recurrent differentiated thyroid cancer [8]. But their cases are mostly papillary carcinoma, and there were only five patients of follicular carcinoma. Our research focused on follicular carcinoma which is rare and more invasive and metastatic compared to papillary carcinoma. Together with previous studies, CyberKnife has demonstrated control of differentiated thyroid cancer with minimal complications.

SBRT for thyroid cancer metastatic lesions can also be treated with c-arm linac. However, if it could also avoid the surrounding organs such as the spine, the vessels, or the lungs, then it would also become a good treatment option.

CyberKnife demonstrated excellent results; however, some of the patients had partial remission or progressive disease. The is because CyberKnife is localized treatment which targets “visible” foci shown by the CT scan. The “invisible” foci may remain and progress. This is a limitation of SBRT. However, some cases reach complete remission over one or two years after repeated CyberKnife treatment for new foci. Since this is a slowly progressive cancer, repeated SBRT can achieve complete remission.

The weakness of CyberKnife treatment is that it is not a systemic treatment but a local targeting therapy. Once metastasized, the cancer cannot be eradicated with CyberKnife. However, it may be adequate for such a slow-progressing cancer just to control the tumor with local targeted therapy.

## Conclusions

Multisession CyberKnife radiosurgery was shown to be successful for the treatment of metastatic and recurrent lesions of follicular thyroid cancer. It successfully controlled this particular slow-progressing cancer to a certain extent. For those patients who cannot tolerate chemotherapy, SBRT with CyberKnife is shown to be a viable option for the treatment of post-surgery recurrent or metastatic follicular thyroid cancer.
